# APOE Genotypes and Alzheimer’s Disease Risk in Older Ugandans

**DOI:** 10.1002/alz.092112

**Published:** 2025-01-03

**Authors:** Kamada Lwere

**Affiliations:** ^1^ Makerere University, Kampala Uganda

## Abstract

**Background:**

Apolipoprotein E (APOE) gene has three common alleles: ԑ2, ԑ3, and ԑ4, which influence Alzheimer’s disease (AD) risk. APOE ԑ4 allele increases AD risk, while APOE ԑ2 allele may protect against AD. This study aimed to examine the distribution and prevalence of APOE genotypes and their association with AD in older Ugandans.

**Method:**

Forty‐three participants aged ≥ 65 years were recruited from two villages in Wakiso district, Uganda. Suspected AD cases were identified using the Montreal Cognitive Assessment (MoCA) tool. APOE genotypes were determined from blood samples using molecular biology techniques.

**Preliminary results:**

The participants (n = 43) had a mean age of 79.0 ± 11.4 years, with a range from 62 to 120 years, and 86% were female. The distribution and prevalence of APOE genotypes varied across the sample, with ԑ3ԑ3 being the most common (53.5%), followed by ԑ2ԑ3 (23.3%), ԑ2ԑ2 (4.7%), and ԑ3ԑ4 (2.3%). The frequency of ԑ3ԑ3 genotype was consistent across different age groups, reflecting its dominance in the studied population. Females displayed a broader variety of APOE genotypes than males, including ԑ2ԑ3, ԑ2ԑ2, and ԑ3ԑ4. APOE ԑ4 allele was more prevalent among participants with higher MoCA scores, suggesting lower AD risk.

**Conclusion:**

This study reveals the APOE genotyping patterns and their relation to AD risk in older Ugandans. The low frequency of APOE ԑ4 allele and its inverse association with AD risk are intriguing and warrant further investigation. Future studies should include larger and more representative samples, as well as other biomarkers and clinical measures of AD.

